# MHC Class IIB Exon 2 Polymorphism in the Grey Partridge (*Perdix perdix*) Is Shaped by Selection, Recombination and Gene Conversion

**DOI:** 10.1371/journal.pone.0069135

**Published:** 2013-07-23

**Authors:** Marta Promerová, Tereza Králová, Anna Bryjová, Tomáš Albrecht, Josef Bryja

**Affiliations:** 1 Institute of Vertebrate Biology, Academy of Sciences of the Czech Republic, Brno, Czech Republic; 2 Department of Medical Biochemistry and Microbiology, Uppsala University, Uppsala, Sweden; 3 Department of Botany and Zoology, Faculty of Science, Masaryk University, Brno, Czech Republic; 4 Department of Zoology, Faculty of Science, Charles University, Prague, Czech Republic; University of Massachusetts, United States of America

## Abstract

Among bird species, the most studied major histocompatibility complex (MHC) is the chicken MHC. Although the number of studies on MHC in free-ranging species is increasing, the knowledge on MHC variation in species closely related to chicken is required to understand the peculiarities of bird MHC evolution. Here we describe the variation of MHC class IIB (MHCIIB) exon 2 in a population of the Grey partridge (*Perdix perdix*), a species of high conservation concern throughout Europe and an emerging galliform model in studies of sexual selection. We found 12 alleles in 108 individuals, but in comparison to other birds surprisingly many sites show signatures of historical positive selection. Individuals displayed between two to four alleles both on genomic and complementary DNA, suggesting the presence of two functional MHCIIB loci. Recombination and gene conversion appear to be involved in generating MHCIIB diversity in the Grey partridge; two recombination breakpoints and several gene conversion events were detected. In phylogenetic analysis of galliform MHCIIB, the Grey partridge alleles do not cluster together, but are scattered through the tree instead. Thus, our results indicate that the Grey partridge MHCIIB is comparable to most other galliforms in terms of copy number and population polymorphism.

## Introduction

Genes of the major histocompatibility complex (MHC) encode for molecules which bind small fragments of peptides and present these on cell surfaces for recognition by immune cells. Recognition of the presented fragment as foreign (i.e. of pathogenic origin), triggers a whole cascade of immune reactions [1], [2]. The evolutionary pressure of parasite burden has been suggested to contribute to the extraordinary polymorphism of MHC genes, which belong among the most variable genes of vertebrate genomes known to date [1], [3], [4]. Apart from pathogen pressure, sexual selection has been shown to influence MHC class I and class II polymorphism by a number of studies in mammals (e.g. [5]–[7]), fish (e.g. [8], [9]), amphibians [10], reptiles [11], [12], and in birds [13]–[20]. The link between MHC polymorphism and mate choice arises because (i) “good” MHC genes confer better viability to offspring [21], [22], or (ii) MHC polymorphism might help avoiding kin mating (reviewed in [23]).

Among birds, the most studied and best described MHC is that of the chicken. It consists of two unlinked clusters of genes: the B-locus and the Y-locus (also called Rfp-Y). Both contain class I and II genes [24]–[26]. However, the B-locus contains highly polymorphic and expressed genes showing strong signals of historical balancing selection, while the Y-locus appears to be less expressed, holds mostly pseudogenes and genes with to date unclear function (e.g. [27]–[32]). The chicken MHC differs from mammalian MHC by the presence of only one dominantly expressed molecule of class I and one of class II, a feature which coined the term ‘minimal essential MHC’ [33], [34]. Other characteristic features of the chicken MHC include its small physical size, dense organisation with short introns, lack of redundancy, and low number of other class I and class II genes with very few pseudogenes [34]–[36]. Some other galliform birds have been shown to share the simple genetic organisation of chicken MHC, such as the pheasant (*Phasianus colchicus*) [37] or black grouse (*Tetrao tetrix*) [32], each with two copies of MHC class IIB (MHCIIB). Other galliforms possess a slightly higher number of loci. For example, there are three MHCIIB loci in turkey (*Meleagris gallopavo*) [38] and several class I and class II genes in the Japanese quail (*Coturnix japonica*), including pseudogenes [39]–[41]. However, similarly to chicken in the Japanese quail only a fraction of the present class I and class II genes are dominantly expressed [39], [40]. In avian species outside the galliforms, the genetic complexity of MHC class I and class II might vary from low copy numbers, e.g. in owls [42], [43] or great snipe (*Gallinago media*) [44], to passerines displaying the most extreme patterns of polymorphism, typically with high levels of gene duplication (e.g. [45]–[50]) and extensive pseudogenes [51]. Although many of the duplicated genes have been shown to transcribe to cDNA (e.g. [49]), little is known about the levels of expression and hence true functional diversity of class I and class II genes in passerines.

It remains unclear whether the compact organization of MHC genes with rather few genes in some birds is ancestral, and how the variability in complexity evolves. The comparison of MHC structure and variation across the galliform birds is therefore necessary in order to analyse the patterns and processes involved in MHC evolution of these birds. Here we studied the structure, variation and evolutionary mechanisms acting on MHCIIB in the Grey partridge (*Perdix perdix*), a galliform bird that is diverged from the chicken by 33.7 million years (based on TimeTree, [52]).

The Grey partridge is a typical farmland inhabitant and game bird. The species used to be common in Central and Eastern Europe, but since the change in agricultural strategy in the 1950 s its populations have been rapidly declining [53], [54]. Our study population originates from the Czech Republic, where the species is bred in captivity for later release of offspring into the wild. Thus, as part of the conservation programme, it is crucial to study adaptive genetic variation in this species and MHC genes have been used as very suitable markers for assessing the status of endangered populations (e.g. [55], [56]). Additionally, the Grey partridge is a socially monogamous species, with females being particularly choosy [57], and, therefore, the MHC markers can be used to test the presence and mechanism of MHC-based mate choice.

## Materials and Methods

### Ethics Statement

The individuals used in this study were sampled in 2010 at a private breeding farm in Letonice, Czech Republic under the permission of the owner. The Grey partridge is not specifically protected in the Czech Republic and all manipulations with animals and methods of sacrifice were approved by the ethic committee of the Academy of Sciences of the Czech Republic (permit number 147/2007 to JB, who is holder of the certificate of competency on Protection Animals against Cruelty, reg. no. V/1/2005/05).

### Study Species, Sampling, DNA and RNA Extraction

The Grey partridge (*Perdix perdix*) is a sedentary, socially monogamous and territorial galliform species bound to open countryside. In total 108 adult individuals (54 females and 54 males, i.e. all breeding pairs in the farm) were sampled prior to nesting, and ca 1 ml of blood was taken by wing venipuncture and stored in 96% ethanol at −20°C until DNA extraction.

Genomic DNA was extracted using the DNeasy Blood & Tissue Kit, Qiagen (Hilden, Germany). Spleen from two sacrificed individuals and blood from additional four individuals were sampled in order to analyse MHC expression. These samples were stored in RNAlater (Qiagen) at −80°C, and RNA was extracted using the RNeasy Plus Mini kit (Qiagen). RNA was transcribed to cDNA using the Transcriptor First Strand cDNA Synthesis kit (Roche) according to the manufacturer’s instructions.

### Design of Species-Specific Primers

In MHCIIB molecules, the amino acids being directly in touch with bound peptides (i.e. the β1 domain) are encoded by exon 2. To obtain initial sequences of Grey partridge MHC we used primers that were published for other galliform species (an overview of tested primers and their positions is given in File S1). We aligned our initial Grey partridge sequence with chicken, ring-necked pheasant, Japanese quail, helmeted guineafowl, black grouse and Indian peafowl and we designed two new primer pairs in conserved regions. For analysis of cDNA, we used primers BLB-fw7126 (5′-GTG CTG GTG GCA CTG CTG G-3′) and BLB-rev7776 (5′-CGT TCT GCA TCA CGT CCG TGG-3′) situated in exon 1 and exon 3, respectively. For high-throughput genotyping on genomic DNA, we designed primers BLBintr1-Fw (5′-TGC CCG CAG CGT TCT TCC TC-3′) and BLBintr2-Rev (5′-TCA CCT TGG GCT CCA CTG CG-3′) situated at the border of intron 1 with exon 2, and the border of intron 2 with exon 3, respectively (File S1).

### MHC Genotyping - PCR, CE-SSCP, Cloning and Sequencing

Grey partridge MHCIIB region displays high GC content, and particularly introns 1 and 3 contain long repetitive GC stretches (data not shown). Thus, we applied PCR chemistry designed for GC-rich target sequences. MHCIIB exon 2 was amplified by PCR in a final volume of 10 µl with the following conditions: 1x Kapa2G GC buffer (including MgCl_2_ in final concentration of 1.5 mM), 0.2 µM of each forward (BLBintr1-Fw) and reverse (BLBintr2-Rev) primer, 0.2 mM mixed dNTPs, 0.5 units of Kapa2G Robust HotStart polymerase (Kapa Biosystems), and a final adjustment with ddH_2_O to 9 µl. 1 µl of DNA (ca 50 ng/µl) was then added to the master-mix. Thermal cycling was performed on the Master-cycler ep (Eppendorf) with an initial denaturation at 94°C for 10 min, followed by 30 cycles of each 94°C (30 sec), 62°C (30 sec) and 72°C (40 sec). A final step of 72°C for 8 min was introduced to finish elongation. PCR products were visualized on agarose gel stained by GoldView™ (Ecoli).

All individuals were genotyped by capillary electrophoresis single strand conformation polymorphism (CE-SSCP) analysis. For this purpose, exon 2 was amplified by PCR as described above, but using fluorescently labelled primers (BLBintr1-Fw: 6-FAM; BLBintr2-Rev: NED). CE-SSCP was performed on a 3130 Genetic Analyzer (Applied Biosystems) as described in [48]. After testing several electrophoresis run temperatures, the best resolution was achieved using a run temperature of 22°C. Electropherograms were analysed in GeneMapper v3.7 (Applied Biosystems).

CE-SSCP displays different alleles by visualizing each sequence variant by a unique shape and position on the electropherogram. In order to obtain nucleotide sequences for all alleles present in the studied population (i.e. all alleles visualized on CE-SSCP), we cloned the amplicons of 24 individuals based on their CE-SSCP genotypes. Amplicons for bacterial cloning were purified using the MinElute PCR purification kit (Qiagen), ligated to the pJET1.2/blunt vector and transformed using the CloneJet™ PCR cloning kit (Fermentas) according to the manufacturer’s instructions. Colonies were screened for inserts of the expected length (cca 400 bp) by PCR (using the vector primers pJET1.2-Fw and pJET1.2-Rev) and gel electrophoresis. Positive clones were then screened using CE-SSCP as described above. In total we screened 220 clones. The CE-SSCP profiles of the clones were compared to the CE-SSCP profiles of individuals’ uncloned amplicons, in order to (i) avoid sequencing of obvious PCR artefacts, (ii) make sure we capture all the alleles of an individual and (iii) avoid multiple repeated sequencing of the same alleles. On average we screened eight clones per individual. For some individuals as much as 23 clones had to be screened before all their alleles displayed by CE-SSCP were captured. Selected clones were then sequenced using the BigDye Terminator Sequencing kit v3.1 (Applied Biosystems) and the primer pJET1.2 Reverse Sequencing Primer (Fermentas).

### Expression Analysis

To verify whether sequences obtained from gDNA using intronic primers are transcribed and hence potentially functional, we analysed cDNA of 6 individuals. The whole MHCIIB exon 2 was amplified using primers BLB-fw7126 (in exon 1) and BLB-rev7776 (in exon 3) with the same PCR conditions as described above. The purified PCR products were then cloned as described above and 3 to 27 clones per individual were Sanger sequenced. Two of these six individuals were screened also for MHCIIB variation at gDNA as described above.

### Analysis of Recombination, Historical Selection, and Gene Conversion

Sequences were edited in BioEdit Sequence Alignment Editor v7.0.5.3 [58] and aligned using the ClustalW algorithm [59]. Primers and intronic sequences were trimmed from clone sequences. As the first nucleotide of the starting amino acid of exon 2 is situated at the end of exon 1, the sequences were cut to 267 bp (two bases in the 5′-end and one base in the 3′-end) to fit the reading frame for further analysis. To avoid analysis of PCR artefacts, we only considered exon 2 variants as ‘true’ alleles if they occurred in at least two individuals or two independent PCRs of the same individual.

Recombination breakpoints were detected using the GARD algorithm in the HyPhy package (available at http://www.datamonkey.org) [60]. Historical selection was analysed by likelihood ratio modelling in the program CodeML, which is a component of the PAML 4 program suite [61]. In this study we compared models allowing for positive selection (M2a and M8) with those assuming no selection (M1a and M7) [62], [63]. The tested models differ in the number and type of included parameters that are based on *ω* ratio, i.e. the ratio of non-synonymous mutations (dN) versus synonymous mutations (dS). Comparison of nested models (M1a with M2a, and M7 with M8, respectively) was obtained using likelihood ratio test statistics; i.e. 2(L_b_-L_a_) was compared with χ^2^ distribution with P_b_-P_a_ degrees of freedom (L_a_, L_b_ – log-likelihood values for each compared model; P_a_, P_b_ – number of parameters for each compared model). The Bayes empirical Bayes (BEB) method was used to calculate posterior probabilities for site classes in models M2a and M8. If the posterior probabilities for some sites are significant (*ω*>1), those sites are inferred to be under positive selection. We also calculated the number of synonymous and non-synonymous substitutions per site, as an overall mean value for all sequences with a standard error estimated by bootstrapping (1000 replicates). The analysis was conducted separately for antigen-binding sites (ABS) and non-ABS derived from the ABS of human HLA-DRB1 [64] in MEGA v5.03 [65].

Occurrence of gene conversion events in MHC IIB exon 2 was tested using GENECONV version 1.81a [66], with 10,000 permutations. The analysis is based on a pairwise comparison of aligned alleles. G-scale value was set to 0, not allowing for any mismatches within the gene conversion tracts. Gene conversion events were considered significant if the global *P* value was lower than 0.05.

### Phylogenetic Analysis

To analyze the relationships among exon 2 sequences of the Grey partridge and other galliform species, we downloaded available MHCIIB and MHCIIY sequences of 14 and five galliform species, respectively, from GenBank (File S2). We used the MHCIIB (DAB1) sequence of the barn owl (GenBank acc. no. EF641260) [42] as outgroup. Phylogenetic analysis of the alignment was performed by maximum likelihood (ML) approach using the PhyML 3.0 online web server [67]. The BIONJ distance-based tree was used as the starting tree and GTR+G as the substitution model. Clade support was tested by 1,000 bootstraps.

## Results

### MHCIIB Intra-Population Variation and Expression

**Figure 1:**
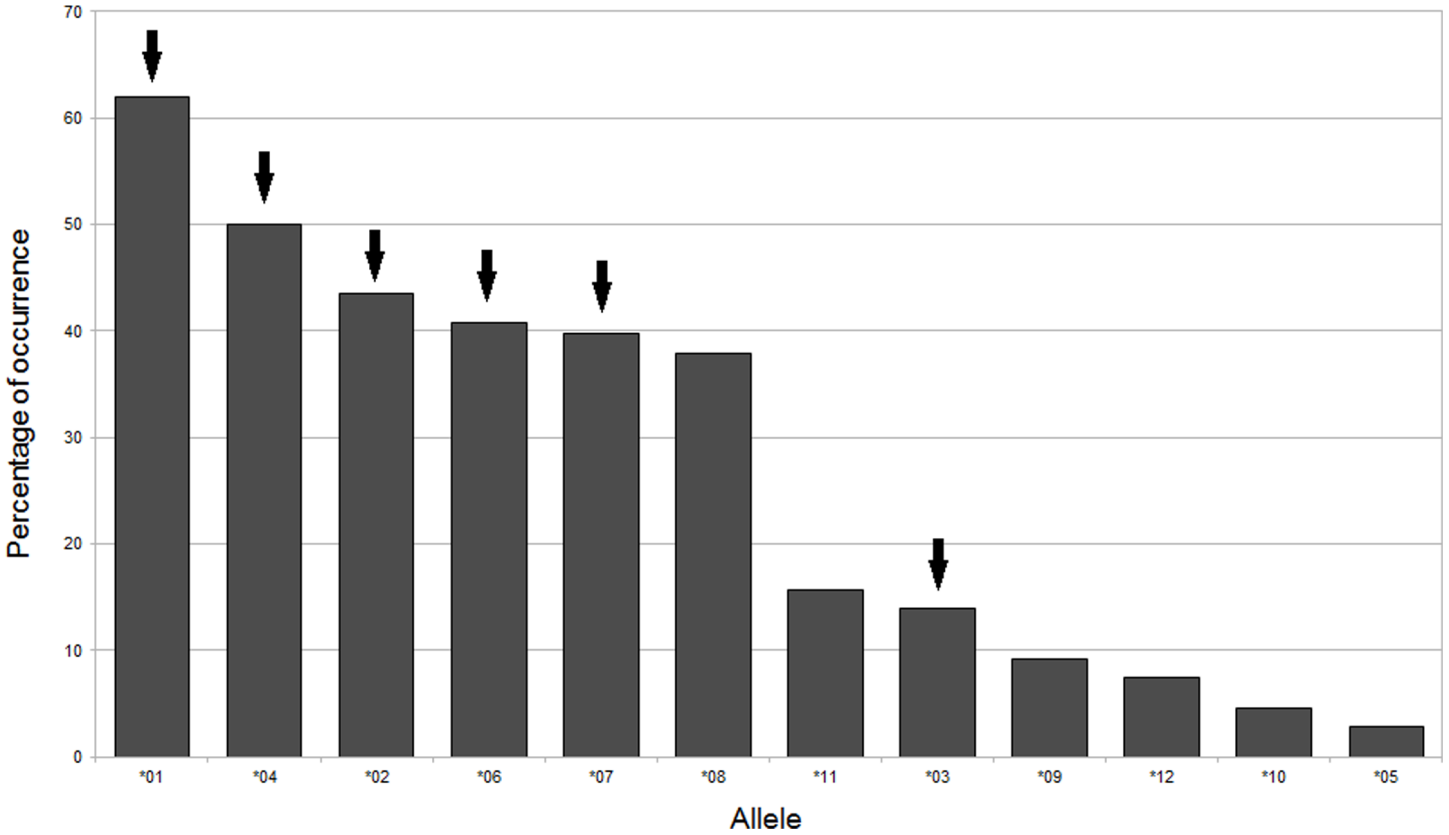
Frequency of occurrence (in %) of MHCIIB exon 2 alleles in 108 individuals, sorted from highest to lowest values. Arrows indicate the alleles found in gDNA as well as cDNA.

In total, 12 different exon 2 variants were identified using CE-SSCP, cloning, and sequencing (sequences are deposited in GenBank, under accession numbers: KF007890-KF007901). Individuals displayed between two to four variants on gDNA. Of the 108 Grey partridge individuals, 17 displayed two alleles, 44 displayed three and 47 displayed four alleles, suggesting the presence of at least two MHCIIB loci in the Grey partridge. The assignment of sequences to specific loci based on phylogenetic analysis was not possible and, for simplicity, we thus call MHCIIB variants as ’alleles‘. In chicken, MHCIIB is called BLB. However, we cannot identify whether the genes we amplify in the Grey partridge are orthologous to BLB. Therefore, following the nomenclature suggestions of Klein et al. [68] we call our alleles *MhcPepe*-DAB*01–12, but for simplicity throughout this paper the alleles are referred to as Pepe*01-Pepe*12. The most abundant allele, Pepe*01 was present in 67 individuals out of 108 (62%), and five other alleles were relatively common, occurring in more than one third of the birds (Fig. 1). The rarest allele, Pepe*05, was only detected in 3 individuals.

**Figure 2:**
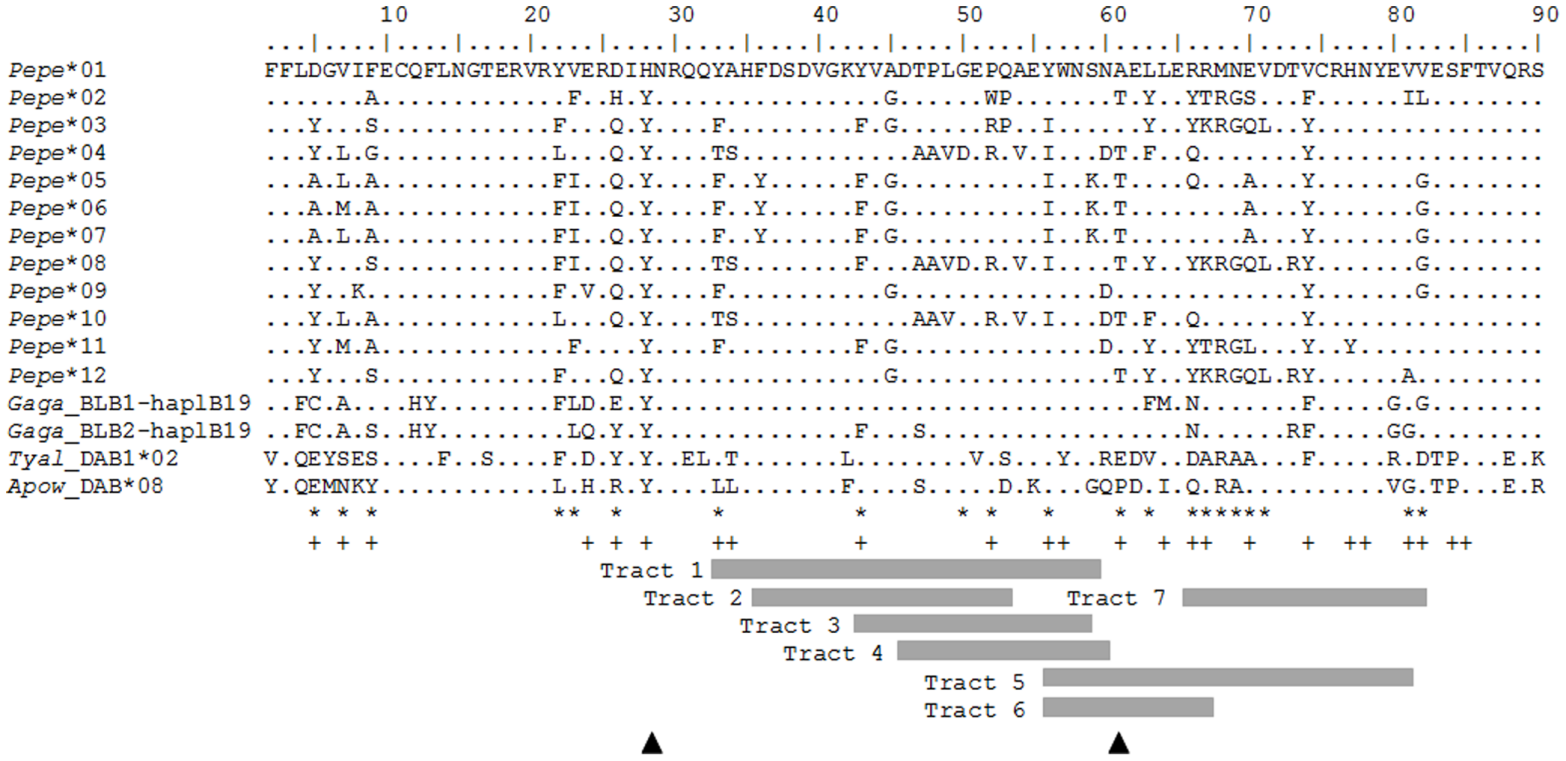
Putative amino acid sequence alignment. The alignment starts at the 2nd amino acid position encoded by the exon 2 in MHCIIB. Sequences of chicken (*Gaga*_BLB1-haplB19 and *Gaga*_BLB2-haplB19, GenBank accession no. AB426151), barn owl (*Tyal*_DAB1-allele02, EU442602) and little spotted kiwi (*Apow*_DAB*allele08, HQ639687) [90] are shown for comparison. Dots represent identity to the sequence on top. Asterisks (*) indicate the amino acid positions under positive selection (based on CodeML analysis, model M8, posterior probability >0.95). Plus signs (+) mark the antigen-binding sites (ABS) in humans [64]. Tracts 1–7 depict codons contained in conversion tracts as given in Table 3. Black triangles represent the placement of two recombination breakpoints as detected by GARD.

All sequences translate into amino-acid sequences without stop codons or indels shifting the reading frame (Fig. 2). Six of the alleles (Pepe*01, *02, *03, *04, *06 and *07) were sequenced also from cDNA of six individuals and cDNA genotypes were identical with the genotypes obtained from genomic DNA of the same individuals, suggesting that both MHCIIB loci are expressed.

### Historical Positive Selection, Recombination and Gene Conversion

Out of the 270 nucleotide positions in exon 2, 63 positions were variable among the 12 alleles. Non-synonymous substitutions per site (dN) outnumbered the synonymous ones (dS) when considering all codons of the sequence (Table 1). Based on the Z-test of positive selection, this difference was significant only for presumable ABS sites (Z-test, Z_dN-dS_ = 2.156, P = 0.017), but not for non-ABS sites (Z-test, Z_dN-dS_ = 0.401, P = 0.344) (Table 1).

**Table 1:** The number of synonymous (dS) and non-synonymous (dN) substitutions per site for ABS, non-ABS and all sites in exon 2 of MHC class IIB (mean±SE); the value of Z-test of positive selection and probability (*P*) that positive selection acts on these sites.

	dN±SE	dS±SE	dN/dS	Z-test	*P*
ABS	0.303±0.075	0.134±0.058	2.261	2.156	0.017
non-ABS	0.066±0.017	0.056±0.025	1.179	0.401	0.344
all sites	0.120±0.020	0.078±0.023	1.538	1.610	0.055

The analysis was conducted using the Nei-Gojobori model [93] with Jukes-Cantor correction. Standard errors were estimated by bootstrap method with 1000 replications. Z-test of positive selection was conducted in MEGA v5.03 [65]. *P* is considered significant at 5% level.

Maximum likelihood models also show that positive selection is acting on the MHCIIB in the Grey partridge (Table 2). Both alternative models which take into account the positive selection fitted our data significantly better than the basic models of neutral evolution (M2a versus M1a: *df* = 2, Test statistic = 49.552, p<0.001; M8 versus M7: *df* = 2, Test statistic = 50.382, p<0.001). Model M8 identified 21 positively selected sites (17 with >99% probability and additional 4 sites with >95% probability; Table 2); 13 of the positively selected sites in M8 correspond to the ABS residues known from humans (Table 2, File S3).

**Table 2:** Results of maximum likelihood models for testing historical selection on exon 2 of MHC class IIB in the Grey partridge.

model	*P*	log-likelihood (L)	parameter estimates	positively selected sites
M1a	2	–969.408	*p*_0_ = 0.575, *p*_1_ * = *0.425	not allowed
M2a	4	–944.632	*p*_0_ = 0.573, *p*_1_ = 0.101, *p*_2_ = 0.326, *ω*_2_ = 8.281	*5*, **7**, **9**, **22**, **23**, *26*, **33**, **43**, *50*, **52**, **56**, *61*, **63**, **66**, **67**, *68*, **69**, **70**, *81*, **82**
M7	2	–969.871	*p* = 0.016, *q* = 0.023	not allowed
M8	4	–944.680	*p*_0_ = 0.674, *p*_1_ = 0.326, *p* = 0.187, *q* = 0.617, *ω* = 8.372	**5**, **7**, **9**, **22**, **23**, **26**, **33**, **43**, *50*, **52**, **56**, *61*, **63**, **66**, **67**, **68**, **69**, **70**, *71*, *81*, **82**

The maximum likelihood models were performed in CodeML integrated in PAML package v4 [61]. *P* indicates a number of parameters considered by the model, *ω* is a parameter based on dN/dS ratio (selection parameter), *p*_n_ is a proportion of sites in a specific *ω* site class, *p* and *q* are the indicators of shape of *β* distribution; models M2a and M8 estimated the positively selected sites of exon 2 by Bayes Empirical Bayes procedure [63]. Sites positively selected with probability >99% are in bold, sites with p>95% are in italics, underlined sites correspond to the ABS residues in humans [64].

Test of recombination revealed two breakpoints (at 79 bp and 178 bp; Fig. 2). The evidence was given by AIC_c_ (i.e. the small-sample-size corrected version of Akaike Information Criterion), which achieved an improvement of ∼ 129 points over the model without recombination. Moreover, both sites were tested for topological incongruence and one site (178 bp) proved to be significant (KH-test, P = 0.01).

Gene conversion analysis in GENECONV showed several significant gene conversion events among the Grey partridge MHCIIB exon 2 sequences (summarised in Table 3 and Fig. 2). Conversion tracts vary in size between 37 to 79 bp. Only one of the 12 exon 2 sequences described in this study shares no fragment with other sequences (Pepe*03).

**Table 3:** Gene conversion events among 12 nucleotide sequences of MHCIIB exon 2 in the Grey partridge.

Tract no.	Seq 1	Seq 2	Simulated *P* value	Start	End	Length	No. of Polymorphisms	Total Differences
1	Pepe*09	Pepe*12	0.0033	96	174	79	22	23
	Pepe*02	Pepe*09	0.024	96	150	55	12	33
2	Pepe*07	Pepe*11	0.013	105	155	51	13	32
	Pepe*05	Pepe*11	0.027	105	155	51	13	31
	Pepe*06	Pepe*11	0.027	105	155	51	13	31
3	Pepe*06	Pepe*12	0.0169	126	162	37	14	30
	Pepe*07	Pepe*12	0.0169	126	162	37	14	30
	Pepe*05	Pepe*12	0.0292	126	162	37	14	29
	Pepe*04	Pepe*08	0.0354	126	174	49	18	23
4	Pepe*01	Pepe*12	0.0064	133	177	45	17	27
5	Pepe*08	Pepe*12	0.0006	165	238	74	25	23
6	Pepe*02	Pepe*08	0.0277	165	196	32	11	35
7	Pepe*04	Pepe*09	0.0017	195	241	47	18	28
	Pepe*09	Pepe*10	0.0038	195	241	47	18	27

Gene conversion tracts as identified by GENECONV [66]. Tract no. as depicted in Fig. 2. Simulated *P* value is based on 10,000 permutations; Start and End indicate the first and last nucleotide position of the conversion tract, respectively; Fragment Length is the length of the conversion tract; No. of Polymorphisms is the number of sites within conversion tract which are polymorphic in the alignment of all alleles; Total Differences is the number of mismatches between the two compared sequences. Sequence pairs are sorted based on the starting position of the conversion tract.

### Phylogenetic Analysis of MHCIIB Sequences in Galliform Birds

**Figure 3:**
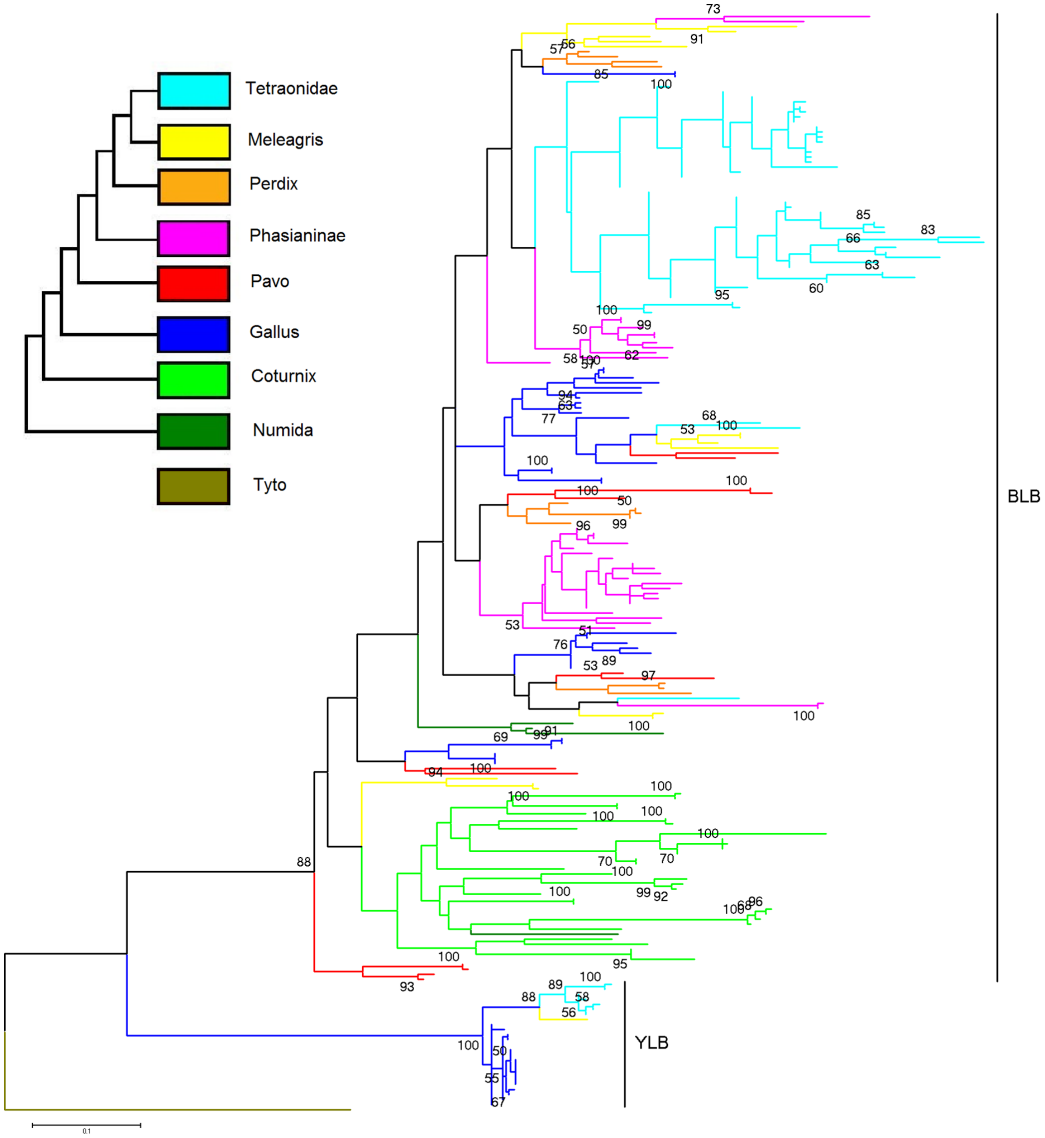
Maximum likelihood tree of exon 2 MHCIIB and MHCIIY sequences across galliform species. Different colours indicate taxa, whose neutral phylogeny is shown in small inserted figure (based on [91] and [92]). The sequence of DAB1 of the barn owl (Acc. no. EF641260) was used as an outgroup. Only bootstrap values >50 are shown. For the complete list of sequences used, with GenBank accession numbers and references see File S2.

In the ML tree of MHC class II sequences of galliform species, the MHCIIB sequences are clearly separated from MHCIIY (Fig. 3). Grey partridge alleles do not cluster together, but are scattered through the tree instead (Fig. 3). Most of the clades, however, are not or only weakly supported, which is probably caused by short length of the sequence and presence of balancing selection as well as concerted evolution typical for avian MHC. It is not possible to assign sequences of exon 2 to specific DAB loci based on the phylogenetic tree.

## Discussion

In this study, we characterized MHCIIB exon 2 variation in a population of the Grey partridge, using an approach combining PCR, cloning, sequencing and CE-SSCP genotyping. We found 12 alleles in total, which is comparable to the MHCIIB diversity in red jungle fowl (*Gallus gallus*), where ten alleles were identified in a captive population of 80 birds [69]. Thus, our results show further evidence that variation in terms of number of alleles per species is lower in galliforms than in other groups of birds. For comparison, 50 alleles were found in 175 great snipes (*Gallinago media*) sampled over several years from a relatively large geographical region [70], 103 alleles at a single locus in 121 lesser kestrels (*Falco naumanni*) from a wide geographical range [71] and 194 exon 2 sequences were found in 237 individuals of collared flycatcher (*Ficedula albicollis*; [51]).

In our study, individuals displayed between two to four alleles. The maximum of four alleles per individual, isolated from both genomic and complementary DNA, and the absence of signatures of pseudogenes (i.e. stop codons or frameshift mutations) suggests the presence of two presumably functional MHCIIB loci in the Grey partridge. This number is equal to the number of known functional MHCIIB genes observed in chicken [34], black grouse [32] and pheasant [37]. Other closely related galliforms display slightly higher number of loci, with turkey (*M. gallopavo*) displaying three [72] and greater prairie-chicken (*Tympanachus cupido*) displaying four MHCIIB loci [73] (Table 4).

**Table 4:** Review of MHC structure in galliform species.

Species	Code	N loci	gDNA	cDNA	Y locus	Gene Names	References
*Coturnix japonica*	*Coja*	7	+	+	?	DAB1, DBB1, DCB1, DDB1,DEB1, DFB1, DGB1	[39]–[41]
*Gallus gallus*	*Gaga*	2	+	+	+	BLB1, BLB2	[24], [26], [34], [40], [69]
*Gallus lafayetii*	*Gala*	?	+	−	?	−	[94]
*Meleagris gallopavo*	*Mega*	3	+	+	+	−	[72], [82]
*Numida meleagris*	*Nume*	?	+	+	?	−	[95]
*Pavo cristatus*	*Pacr*	3	+	−	?	−	[96]
*Perdix perdix*	*Pepe*	2	+	+	?	DAB	this study
*Phasianus colchicus*	*Phco*	2	+	+	+	DAB1, DAB2	[13], [37], [40], [81]
*Tetrao tetrix*	*Tete*	2	+	+	+	−	[32]
*Tympanuchus cupido*	*Tycu*	4	+	−	+	−	[73]

Most galliform species display low number of MHCIIB loci, except the migratory Japanese Quail (*Coturnix japonica*). The second genetically unlinked MHC region called Y locus has been found in half of them. Columns gDNA and cDNA show what kind of template was used in the studies. Species are ordered alphabetically. Used symbols:+yes, - no, ? yet unknown.

CE-SSCP genotyping method has by some studies been shown to underestimate MHC variation [74], [75]. This is particularly true in case of highly duplicated genes where locus-specific amplification is not possible, such as passerine MHC class I [76]. However, the method has been successfully applied in a wide range of species where MHC class I and class II display moderate levels of polymorphism, such as mammals (e.g. [56], [77], [78]) and galliform birds [79]. While studies using next-generation sequencing of MHC in passerines have reported highly duplicated loci with almost 200 alleles of both MHCIIB [80] and MHCI [76] from single populations, such extensive variation is not expected in galliforms. Hence, we believe that the applied method is suitable for MHCIIB genotyping in the Grey partridge, particularly when combined with cloning and Sanger sequencing.

A feature typical for the structure of chicken MHC and some other closely related species is the presence of a second, unlinked cluster of MHC genes, the Y locus [27], [32], [81], [82] (Table 4). This cluster consists of genes with yet unclear functional significance [32], [81], but some of the MHC genes situated in the Y region have been shown to be expressed [31], [82], [83]. We tested many combinations of primers, including those that specifically amplify Y loci in other species [32], but were unable to isolate sequences of Y loci in the present study (Fig. 3). It is possible that if the Grey partridge, like several closely related species, has a Y locus, it is distinct enough in sequence such that it is not amplified with any of the primers used here. More powerful approaches, such as whole-genome sequencing, or screening of BAC libraries might be necessary to rule out the presence of Y loci in the Grey partridge (see for example [84], where only whole genome sequencing confirmed the complete absence of MHC class II in the cod).

Positive selection and gene conversion appear to have prominent roles in the evolution of MHCIIB diversity in the Grey partridge. We identified widespread traces of positive selection in exon 2, with 21 out of 89 tested sites showing *ω* significantly higher than 1. This level of historical positive selection is extraordinarily high compared to other studies of avian species analysed by the same methods. For example in the barn owl (*T. alba*) seven sites showed signs of positive selection [42], 12 sites in the blue petrel (*Halobaena caerulea*) [85], and 10 sites in the house sparrow (*Passer domesticus*) [86]. Moreover, recombination and gene conversion were shown to significantly affect sequence diversity in the Grey partridge. Fifteen out of 21 sites inferred to be under positive selection lay within conversion tracts (Fig. 2). Gene conversion processes within the MHC create polymorphism much faster than point mutations by reshuffling polymorphic fragments among alleles, thus creating new haplotypes [87], [88]. Variation at avian MHC has been shown to originate at least partially from gene conversion (e.g. [37]). Gene conversion can be an important driver of allelic diversity especially in populations that have undergone bottleneck and suffered from depleted MHC variation. In one such population of the Berthelot’s pipit, gene conversion created diversity by one order of magnitude faster than generated by point mutations [89]. It is highly probable that the processes of gene conversion, recombination and historical selection all act in concert, and their respective contribution to the generation and maintenance of sequence diversity thus might often be difficult to tell apart.

Our study showed that even the captive Grey partridge population display MHCIIB variation that is comparable to other galliform species. The European population of this species decreased by more than 80% since 1980 [54] and conservation programs are often using captive animals to support natural populations. Here we provide first data showing that the studied captive population could have a good chance to withstand pathogens after release. Results of this study will also be useful in testing the alternative hypotheses of MHC-based mate choice in the Grey partridge, which is an emerging model in the research of sexual selection [57].

## Supporting Information

File S1 List of all primers tested for PCR amplification of MHCIIB in the Grey partridge and their schematic overview.
(DOC)

File S2 Overview of all MHCIIB sequences of Galliformes used in the phylogenetic analysis, with references and GenBank accession numbers.
(DOC)

File S3 Posterior means of ω for each codon of MHCIIB exon 2 in the Grey partridge, calculated as the average of ω over the 11 site classes, weighted by the posterior probabilities under the random-sites model M8 (β and ω). The posterior probabilities were computed by the Bayes empirical Bayes procedure in the program CodeML implemented in the PAML3.14 package.
(DOCX)
