# Supplementary material for: MHC Class IIB Exon 2 Polymorphism in the Grey Partridge (Perdix perdix) Is Shaped by Selection, Recombination and Gene Conversion
Source: PLoS One. 2013 Jul 23;8(7):e69135. doi: 10.1371/journal.pone.0069135 (PMC3720538; doi:10.1371/journal.pone.0069135)
Supplement: File S1 — List of all primers tested for PCR amplification of MHCIIB in the Grey partridge and their schematic overview. (DOC) [file pone.0069135.s001.doc]

**Supporting Information S1: List of all primers tested for PCR amplification of MHC IIB in the Grey Partridge and their schematic overview**

**Article title**: MHC class IIB exon 2 polymorphism in the Grey partridge (*Perdix perdix*) is shaped by selection, recombination and gene conversion

**Journal name**: PLOS ONE

**Authors**: Promerová M., Králová T., Bryjová A., Albrecht T. and Bryja J.

**Corresponding author**: Josef Bryja; e-mail: bryja@brno.cas.cz

(a) Schematic overview of partial MHC Class IIB gene in the Grey partridge with positions of all tested primers.

**
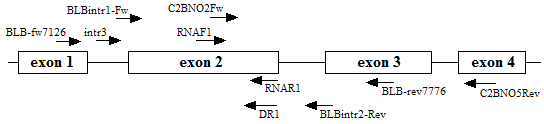
**

(b) List of the primers tested for PCR amplification of MHC class IIB in the Grey Partridge.

| **primer name** | **primer sequence (5' → 3')** | **designed for** | **reference** |
| --- | --- | --- | --- |
| RNAF1 | GACAGCGAAGTGGGGAAATA | *Tetrao tetrix* | Strand et al. 2007 |
| RNAR1 | CGCTCCTCTGCACCGTGA | *Tetrao tetrix* | Strand et al. 2007 |
| C2BNO2Fw | GAGTGCCACTACCTGAACGGCACCGAGG | *Coturnix japonica* | Hosomichi et al. 2006 |
| C2BNO5Rev | GCGCCAGGAAGACGAGCCCCAGCAC | *Coturnix japonica* | Hosomichi et al. 2006 |
| intr3 | GCGGCTGTGTGCCTGACC | *Phasianus colchicus* | Wittzell et al. 1994 |
| DR1 | GCTCCTCTGCACCGTGAAGGA | *Phasianus colchicus* | Wittzell et al. 1994 |
| BLB-fw7126 | GTGCTGGTGGCACTGCTGG | *Perdix perdix* | this study |
| BLB-rev7776 | CGTTCTGCATCACGTCCGTGG | *Perdix perdix* | this study |
| BLBintr1-Fw | TGCCCGCAGCGTTCTTCCTC | *Perdix perdix* | this study |
| BLBintr2-Rev | TCACCTTGGGCTCCACTGCG | *Perdix perdix* | this study |

**References:**

Hosomichi K, Shiina T, Suzuki S, Tanaka M, Shimizu S, Iwamoto S, Hara H, Yoshida Y, Kulski JK, Inoko H, Hanzawa K (2006) The major histocompatibility complex (Mhc) class IIB region has greater genomic structural flexibility and diversity in the quail than the chicken. BMC Genomics 7: 322–335.

Strand T, Westerdahl H, Höglund J, Alatalo RV, Siitari H (2007) The Mhc class II of the Black grouse (*Tetrao tetrix*) consists of low numbers of B and Y genes with variable diversity and expression. Immunogenetics 59: 725–734.

Wittzell H, VonSchantz T, Zoorob R, Auffray C (1994) Molecular characterization of 3 MHC Class II B haplotypes in the ring-necked pheasant. Immunogenetics 39: 395-403.
