# Supplementary material for: MHC Class IIB Exon 2 Polymorphism in the Grey Partridge (Perdix perdix) Is Shaped by Selection, Recombination and Gene Conversion
Source: PLoS One. 2013 Jul 23;8(7):e69135. doi: 10.1371/journal.pone.0069135 (PMC3720538; doi:10.1371/journal.pone.0069135)
Supplement: File S2 — Overview of all MHCIIB sequences of Galliformes used in the phylogenetic analysis, with references and GenBank accession numbers. (DOC) [file pone.0069135.s002.doc]

**Supporting Information S2: Overview of all MHCIIB sequences of Galliformes used in the phylogenetic analysis, with references and GenBank accession numbers**

**Article title**: MHC class IIB exon 2 polymorphism in the Grey partridge (*Perdix perdix*) is shaped by selection, recombination and gene conversion

**Journal name**: PLOS ONE

**Authors**: Promerová M., Králová T., Bryjová A., Albrecht T. and Bryja J.

**Corresponding author**: Josef Bryja; e-mail: bryja@brno.cas.cz

| **ID** | **Species** | **Abbreviation** | **English name** | **GenBank Accesion no.** | **Ref.** |
| --- | --- | --- | --- | --- | --- |
| **BLB sequences** | | | | | |
| 1 | *Chrysolophus amherstiae* | Cham | Lady Amherst's Pheasant | JQ001777 | 1 |
| 2 | *Syrmaticus reevesii* | Syre | Reeves's Pheasant | JQ001778 | 1 |
| 3 | *Crossoptilon crossoptilon* | Crcr | White Eared Pheasant | JQ001779 | 1 |
| 4 | *Crossoptilon auritum* | Crau | Blue Eared Pheasant | JQ001781 | 1 |
| 5 | *Crossoptilon auritum* | Crau | Blue Eared Pheasant | JQ001782 | 1 |
| 6 | *Bonasa bonasia* | Bobo | Hazel Grouse | GQ851944 | 2 |
| 7 | *Bonasa bonasia* | Bobo | Hazel Grouse | GQ851945 | 2 |
| 8 | *Bonasa bonasia* | Bobo | Hazel Grouse | GQ851943 | 2 |
| 9 | *Gallus lafayettei* | Gala | Ceylon jungle fowl | AY839724 | 3 |
| 10 | *Gallus lafayettei* | Gala | Ceylon jungle fowl | DQ017586 | 3 |
| 11 | *Gallus lafayettei* | Gala | Ceylon jungle fowl | AY839722 | 3 |
| 12 | *Gallus lafayettei* | Gala | Ceylon jungle fowl | AY839723 | 3 |
| 13 | *Numida meleagris* | Nume | Helmeted Guineafowl | EU826065 | 4 |
| 14 | *Numida meleagris* | Nume | Helmeted Guineafowl | EU826064 | 4 |
| 15 | *Numida meleagris* | Nume | Helmeted Guineafowl | EU030445 | 5 |
| 16 | *Numida meleagris* | Nume | Helmeted Guineafowl | EF643464 | 6 |
| 17 | *Pavo cristatus* | Pacr | Indian Peafowl | JQ001780 | 1 |
| 18 | *Pavo cristatus* | Pacr | Indian Peafowl | AY928104 | 7 |
| 19 | *Pavo cristatus* | Pacr | Indian Peafowl | AY928102 | 7 |
| 20 | *Pavo cristatus* | Pacr | Indian Peafowl | AY928100 | 7 |
| 21 | *Pavo cristatus* | Pacr | Indian Peafowl | AY928098 | 7 |
| 22 | *Pavo cristatus* | Pacr | Indian Peafowl | AY928096 | 7 |
| 23 | *Pavo cristatus* | Pacr | Indian Peafowl | AY928094 | 7 |
| 24 | *Pavo cristatus* | Pacr | Indian Peafowl | AY928103 | 7 |
| 25 | *Pavo cristatus* | Pacr | Indian Peafowl | AY928101 | 7 |
| 26 | *Pavo cristatus* | Pacr | Indian Peafowl | AY928099 | 7 |
| 27 | *Pavo cristatus* | Pacr | Indian Peafowl | AY928097 | 7 |
| 28 | *Pavo cristatus* | Pacr | Indian Peafowl | AY928095 | 7 |
| 29 | *Pavo cristatus* | Pacr | Indian Peafowl | AY928093 | 7 |
| 30 | *Meleagris gallopavo* | Mega | Wild Turkey | AM233872 | 8 |
| 31 | *Meleagris gallopavo* | Mega | Wild Turkey | AM233486 | 8 |
| 32 | *Meleagris gallopavo* | Mega | Wild Turkey | FJ951623 | 9 |
| 33 | *Meleagris gallopavo* | Mega | Wild Turkey | GU189291 | 10 |
| 34 | *Meleagris gallopavo* | Mega | Wild Turkey | GU189289 | 10 |
| 35 | *Meleagris gallopavo* | Mega | Wild Turkey | GU189287 | 10 |
| 36 | *Meleagris gallopavo* | Mega | Wild Turkey | GU189285 | 10 |
| 37 | *Meleagris gallopavo* | Mega | Wild Turkey | GU189283 | 10 |
| 38 | *Meleagris gallopavo* | Mega | Wild Turkey | GU189288 | 10 |
| 39 | *Meleagris gallopavo* | Mega | Wild Turkey | GU189286 | 10 |
| 40 | *Meleagris gallopavo* | Mega | Wild Turkey | GU189284 | 10 |
| 41 | *Meleagris gallopavo* | Mega | Wild Turkey | DQ993255 | 11 |
| 42 | *Tympanuchus cupido* | Tycu | Greater Prairie Chicken | HM011586 | 12 |
| 43 | *Tympanuchus cupido* | Tycu | Greater Prairie Chicken | HM011584 | 12 |
| 44 | *Tympanuchus cupido* | Tycu | Greater Prairie Chicken | HM011582 | 12 |
| 45 | *Tympanuchus cupido* | Tycu | Greater Prairie Chicken | HM011580 | 12 |
| 46 | *Tympanuchus cupido* | Tycu | Greater Prairie Chicken | HM011578 | 12 |
| 47 | *Tympanuchus cupido* | Tycu | Greater Prairie Chicken | HM011576 | 12 |
| 48 | *Tympanuchus cupido* | Tycu | Greater Prairie Chicken | HM011574 | 12 |
| 49 | *Tympanuchus cupido* | Tycu | Greater Prairie Chicken | HM011585 | 12 |
| 50 | *Tympanuchus cupido* | Tycu | Greater Prairie Chicken | HM011583 | 12 |
| 51 | *Tympanuchus cupido* | Tycu | Greater Prairie Chicken | HM011581 | 12 |
| 52 | *Tympanuchus cupido* | Tycu | Greater Prairie Chicken | HM011579 | 12 |
| 53 | *Tympanuchus cupido* | Tycu | Greater Prairie Chicken | HM011577 | 12 |
| 54 | *Tympanuchus cupido* | Tycu | Greater Prairie Chicken | HM011575 | 12 |
| 55 | *Tympanuchus cupido* | Tycu | Greater Prairie Chicken | HM011573 | 12 |
| 56 | *Tympanuchus cupido* | Tycu | Greater Prairie Chicken | FJ232515 | 13 |
| 57 | *Tympanuchus cupido* | Tycu | Greater Prairie Chicken | FJ232509 | 13 |
| 58 | *Tympanuchus cupido* | Tycu | Greater Prairie Chicken | FJ232510 | 13 |
| 59 | *Tympanuchus cupido* | Tycu | Greater Prairie Chicken | GQ176850 | 14 |
| 60 | *Tympanuchus cupido* | Tycu | Greater Prairie Chicken | GQ176848 | 14 |
| 61 | *Tympanuchus cupido* | Tycu | Greater Prairie Chicken | FJ232511 | 14 |
| 62 | *Tympanuchus cupido* | Tycu | Greater Prairie Chicken | GQ176851 | 14 |
| 63 | *Tympanuchus cupido* | Tycu | Greater Prairie Chicken | GQ176849 | 14 |
| 64 | *Tympanuchus cupido* | Tycu | Greater Prairie Chicken | FJ232517 | 14 |
| 65 | *Tympanuchus cupido* | Tycu | Greater Prairie Chicken | FJ232514 | 14 |
| 66 | *Tympanuchus cupido* | Tycu | Greater Prairie Chicken | FJ232512 | 14 |
| 67 | *Tympanuchus cupido* | Tycu | Greater Prairie Chicken | FJ232518 | 14 |
| 68 | *Tympanuchus cupido* | Tycu | Greater Prairie Chicken | FJ232516 | 14 |
| 69 | *Tympanuchus cupido* | Tycu | Greater Prairie Chicken | FJ232513 | 14 |
| 70 | *Tetrao tetrix* | Tete | Black Grouse | JF509706 | 15 |
| 71 | *Tetrao tetrix* | Tete | Black Grouse | EF174544 | 16 |
| 72 | *Tetrao tetrix* | Tete | Black Grouse | EF174546 | 16 |
| 73 | *Tetrao tetrix* | Tete | Black Grouse | HQ108384 | 15 |
| 74 | *Tetrao tetrix* | Tete | Black Grouse | HQ108382 | 15 |
| 75 | *Tetrao tetrix* | Tete | Black Grouse | HQ108380 | 15 |
| 76 | *Tetrao tetrix* | Tete | Black Grouse | JF509707 | 15 |
| 77 | *Tetrao tetrix* | Tete | Black Grouse | HQ108385 | 15 |
| 78 | *Tetrao tetrix* | Tete | Black Grouse | HQ108383 | 15 |
| 79 | *Tetrao tetrix* | Tete | Black Grouse | HQ108381 | 15 |
| 80 | *Tetrao tetrix* | Tete | Black Grouse | GQ181215 | 2 |
| 81 | *Tetrao tetrix* | Tete | Black Grouse | GQ181214 | 2 |
| 82 | *Tetrao tetrix* | Tete | Black Grouse | EF174551 | 16 |
| 83 | *Tetrao tetrix* | Tete | Black Grouse | EF174549 | 16 |
| 84 | *Tetrao tetrix* | Tete | Black Grouse | EF174547 | 16 |
| 85 | *Tetrao tetrix* | Tete | Black Grouse | EF174545 | 16 |
| 86 | *Tetrao tetrix* | Tete | Black Grouse | EF174552 | 16 |
| 87 | *Tetrao tetrix* | Tete | Black Grouse | EF174550 | 16 |
| 88 | *Tetrao tetrix* | Tete | Black Grouse | EF174548 | 16 |
| 89 | *Phasianus colchicus* | Phco | Common Pheasant | EF211109 | 17 |
| 90 | *Phasianus colchicus* | Phco | Common Pheasant | EF211107 | 17 |
| 91 | *Phasianus colchicus* | Phco | Common Pheasant | EF211105 | 17 |
| 92 | *Phasianus colchicus* | Phco | Common Pheasant | EF211103 | 17 |
| 93 | *Phasianus colchicus* | Phco | Common Pheasant | EF211108 | 17 |
| 94 | *Phasianus colchicus* | Phco | Common Pheasant | EF211106 | 17 |
| 95 | *Phasianus colchicus* | Phco | Common Pheasant | EF211104 | 17 |
| 96 | *Phasianus colchicus* | Phco | Common Pheasant | HQ738665 | 18 |
| 97 | *Phasianus colchicus* | Phco | Common Pheasant | HQ738663 | 18 |
| 98 | *Phasianus colchicus* | Phco | Common Pheasant | HQ738661 | 18 |
| 99 | *Phasianus colchicus* | Phco | Common Pheasant | HQ738659 | 18 |
| 100 | *Phasianus colchicus* | Phco | Common Pheasant | HQ738657 | 18 |
| 101 | *Phasianus colchicus* | Phco | Common Pheasant | HQ738655 | 18 |
| 102 | *Phasianus colchicus* | Phco | Common Pheasant | HQ738664 | 18 |
| 103 | *Phasianus colchicus* | Phco | Common Pheasant | HQ738662 | 18 |
| 104 | *Phasianus colchicus* | Phco | Common Pheasant | HQ738660 | 18 |
| 105 | *Phasianus colchicus* | Phco | Common Pheasant | HQ738658 | 18 |
| 106 | *Phasianus colchicus* | Phco | Common Pheasant | HQ738656 | 18 |
| 107 | *Phasianus colchicus* | Phco | Common Pheasant | AJ224352 | 19 |
| 108 | *Phasianus colchicus* | Phco | Common Pheasant | AJ224351 | 19 |
| 109 | *Phasianus colchicus* | Phco | Common Pheasant | AJ224347 | 19 |
| 110 | *Phasianus colchicus* | Phco | Common Pheasant | X75406 | 20 |
| 111 | *Phasianus colchicus* | Phco | Common Pheasant | AJ224350 | 19 |
| 112 | *Phasianus colchicus* | Phco | Common Pheasant | AJ224346 | 19 |
| 113 | *Phasianus colchicus* | Phco | Common Pheasant | X75407 | 20 |
| 114 | *Phasianus colchicus* | Phco | Common Pheasant | X75405 | 20 |
| 115 | *Phasianus colchicus* | Phco | Common Pheasant | X75403 | 20 |
| 116 | *Phasianus colchicus* | Phco | Common Pheasant | AJ224349 | 19 |
| 117 | *Phasianus colchicus* | Phco | Common Pheasant | AJ224348 | 19 |
| 118 | *Phasianus colchicus* | Phco | Common Pheasant | AJ224344 | 19 |
| 119 | *Phasianus colchicus* | Phco | Common Pheasant | AJ224345 | 19 |
| 120 | *Coturnix japonica* | Coja | Japanese Quail | AB265805 | 21 |
| 121 | *Coturnix japonica* | Coja | Japanese Quail | AB265803 | 21 |
| 122 | *Coturnix japonica* | Coja | Japanese Quail | AB282651 | 21 |
| 123 | *Coturnix japonica* | Coja | Japanese Quail | AB282649 | 21 |
| 124 | *Coturnix japonica* | Coja | Japanese Quail | AB282647 | 21 |
| 125 | *Coturnix japonica* | Coja | Japanese Quail | AB265804 | 21 |
| 126 | *Coturnix japonica* | Coja | Japanese Quail | AB282650 | 21 |
| 127 | *Coturnix japonica* | Coja | Japanese Quail | AB282648 | 21 |
| 128 | *Coturnix japonica* | Coja | Japanese Quail | AB264282 | 21 |
| 129 | *Coturnix japonica* | Coja | Japanese Quail | AB181876 | 21 |
| 130 | *Coturnix japonica* | Coja | Japanese Quail | AB181872 | 21 |
| 131 | *Coturnix japonica* | Coja | Japanese Quail | AB181870 | 21 |
| 132 | *Coturnix japonica* | Coja | Japanese Quail | AB181868 | 21 |
| 133 | *Coturnix japonica* | Coja | Japanese Quail | AB181866 | 21 |
| 134 | *Coturnix japonica* | Coja | Japanese Quail | AB181863 | 21 |
| 135 | *Coturnix japonica* | Coja | Japanese Quail | AB181861 | 21 |
| 136 | *Coturnix japonica* | Coja | Japanese Quail | AB181869 | 21 |
| 137 | *Coturnix japonica* | Coja | Japanese Quail | AB181867 | 21 |
| 138 | *Coturnix japonica* | Coja | Japanese Quail | AB181865 | 21 |
| 139 | *Coturnix japonica* | Coja | Japanese Quail | AB110481 | 22 |
| 140 | *Coturnix japonica* | Coja | Japanese Quail | AB110479 | 22 |
| 141 | *Coturnix japonica* | Coja | Japanese Quail | AB110469 | 22 |
| 142 | *Coturnix japonica* | Coja | Japanese Quail | AB110467 | 22 |
| 143 | *Coturnix japonica* | Coja | Japanese Quail | AB110480 | 22 |
| 144 | *Coturnix japonica* | Coja | Japanese Quail | AB110474 | 22 |
| 145 | *Coturnix japonica* | Coja | Japanese Quail | AB110470 | 22 |
| 146 | *Coturnix japonica* | Coja | Japanese Quail | AB110468 | 22 |
| 147 | *Coturnix japonica* | Coja | Japanese Quail | AB110466 | 22 |
| 148 | *Coturnix japonica* | Coja | Japanese Quail | AB181878 | 23 |
| 149 | *Coturnix japonica* | Coja | Japanese Quail | AB181875 | 23 |
| 150 | *Coturnix japonica* | Coja | Japanese Quail | AB181864 | 23 |
| 151 | *Coturnix japonica* | Coja | Japanese Quail | AB181877 | 23 |
| 152 | *Coturnix japonica* | Coja | Japanese Quail | AB181873 | 23 |
| 153 | *Gallus gallus* | Gaga | Chicken | AB426154 | 24 |
| 154 | *Gallus gallus* | Gaga | Chicken | AB426152 | 24 |
| 155 | *Gallus gallus* | Gaga | Chicken | AB426150 | 24 |
| 156 | *Gallus gallus* | Gaga | Chicken | AB426148 | 24 |
| 157 | *Gallus gallus* | Gaga | Chicken | AB426146 | 24 |
| 158 | *Gallus gallus* | Gaga | Chicken | AB426144 | 24 |
| 159 | *Gallus gallus* | Gaga | Chicken | AB426142 | 24 |
| 160 | *Gallus gallus* | Gaga | Chicken | AB426153 | 24 |
| 161 | *Gallus gallus* | Gaga | Chicken | AB426151 | 24 |
| 162 | *Gallus gallus* | Gaga | Chicken | AB426149 | 24 |
| 163 | *Gallus gallus* | Gaga | Chicken | AB426147 | 24 |
| 164 | *Gallus gallus* | Gaga | Chicken | AB426145 | 24 |
| 165 | *Gallus gallus* | Gaga | Chicken | AB426143 | 24 |
| 167 | *Gallus gallus* | Gaga | Chicken | AB426141 | 24 |
| 168 | *Gallus gallus* | Gaga | Chicken | AB268588 | 25 |
| 169 | *Tyto alba* | Tyal | Barn Owl | EF641260 | 26 |
| **YLB sequences** | | | | | |
| 170 | *Gallus gallus* | Gaga | Chicken | AB020220 | 27 |
| 171 | *Gallus gallus* | Gaga | Chicken | AB020221 | 27 |
| 172 | *Gallus gallus* | Gaga | Chicken | AB020322 | 27 |
| 173 | *Gallus gallus* | Gaga | Chicken | AB020323 | 27 |
| 174 | *Gallus gallus* | Gaga | Chicken | AB020324 | 27 |
| 175 | *Gallus gallus* | Gaga | Chicken | AB020325 | 27 |
| 176 | *Gallus gallus* | Gaga | Chicken | AB020326 | 27 |
| 177 | *Gallus gallus* | Gaga | Chicken | AB020327 | 27 |
| 178 | *Gallus gallus* | Gaga | Chicken | AB020328 | 27 |
| 179 | *Gallus gallus* | Gaga | Chicken | AB020329 | 27 |
| 180 | *Gallus gallus* | Gaga | Chicken | AB020330 | 27 |
| 181 | *Gallus gallus* | Gaga | Chicken | AB020331 | 27 |
| 182 | *Gallus gallus* | Gaga | Chicken | AB020332 | 27 |
| 183 | *Coturnix coturnix* | Coco | Common Quail | AB020333 | 27 |
| 184 | *Gallus gallus* | Gaga | Chicken | AF452564 | 28 |
| 185 | *Gallus gallus* | Gaga | Chicken | AF452565 | 28 |
| 186 | *Gallus gallus* | Gaga | Chicken | AF452566 | 28 |
| 187 | *Tetrao tetrix* | Tete | Black Grouse | EF174539 | 16 |
| 188 | *Tetrao tetrix* | Tete | Black Grouse | EF174540 | 16 |
| 189 | *Tetrao tetrix* | Tete | Black Grouse | EF174541 | 16 |
| 190 | *Tetrao tetrix* | Tete | Black Grouse | EF174542 | 16 |
| 191 | *Tetrao tetrix* | Tete | Black Grouse | EF174543 | 16 |
| 192 | *Tympanuchus cupido* | Tycu | Greater Prairie Chicken | FJ232519 | 14 |
| 193 | *Tympanuchus cupido* | Tycu | Greater Prairie Chicken | FJ232520 | 14 |

**References:**

1. Zhang Y, Zou F (unpubl.) Cloning and Sequence Analysis of the MHC B-LB Gene in Five Pheasants.
2. Strand TM, Hoglund J (unpubl.) RSCA-based MHC class II B typing of black grouse families
3. Lambourne MD, Si W, Niemiec PK, Read LR, Kariyawasam S, Sharif S (2005) Identification of novel polymorphisms in the B-LB locus of Gallus lafayettei. Anim Genet 36(5): 445-448.
4. Kumar S, Mehra S, Shukla S, Mehra M, Gupta A, Sharma D (unpubl.) Genetic polymorphism in BLB2 gene between guinea fowl and other poultry species
5. Kumar S, Mehra S, Sharma D (unpubl.) Characterization of BLB2 in guinea fowl
6. Kumar S, Mehra S, Sharma D (unpubl.) Genetic polymorphism in BLB2 gene between guinea fowl and other poultry species
7. Hale ML, Verduijn MH, Moller AP, Wolff K, Petrie M (2009) Is the peacock's train an honest signal of genetic quality at the major histocompatibility complex? J Evol Biol 22: 1284-1294.
8. Ahmed KA, Saxena VK, Saxena M, Ara A, Pramod AB, Rajaram ML, Dorman KS, Majumdar S, Rasool TJ (2007) Molecular cloning and sequencing of MHC class II beta 1 domain of turkey reveals high sequence identity with chicken. Int J Immunogenet 34(2): 97-105.
9. Chaves LD, Krueth SB, Reed KM (unpubl.) Defining the Turkey MHC: Sequence and Genes of the B-Locus
10. Chaves LD, Faile GM, Krueth SB, Hendrickson JA, Reed KM (2010) Haplotype variation, recombination, and gene conversion within the turkey MHC-B locus. Immunogenetics 62(7): 465-477.
11. Chaves LD, Krueth SB, Reed KM (2009) Defining the turkey MHC: sequence and genes of the B locus. J Immunol 183(10): 6530-6537.
12. Eimes JA, Bollmer JL, Whittingham LA, Johnson JA, VAN Oosterhout C, Dunn PO (2011) Rapid loss of MHC class II variation in a bottlenecked population is explained by drift and loss of copy number variation. J Evol Biol 24(9): 1847-1856.
13. Eimes JA, Bollmer JL, Dunn PO, Whittingham LA, Wimpee C (direct submission)
14. Eimes JA, Bollmer JL, Dunn PO, Whittingham LA, Wimpee C (2010) Mhc class II diversity and balancing selection in greater prairie-chickens. Genetica 138(2): 265-271.
15. Strand T, Segelbacher G, Quintela M, Xiao L, Axelsson T, Höglund J (2012) Can balancing selection on MHC loci counteract genetic drift in small fragmented populations of black grouse? Ecol Evol 2: 341-353.
16. Strand T, Westerdahl H, Hoglund J, V Alatalo R, Siitari H (2007) The Mhc class II of the Black grouse (Tetrao tetrix) consists of low numbers of B and Y genes with variable diversity and expression. Immunogenetics 59(9): 725-734.
17. Baratti M, Ammannati M, Magnelli C, Massolo A, Dessi-Fulgheri F (2010) Are large wattles related to particular MHC genotypes in the male pheasant? Genetica 138(6): 657-665.
18. Baratti M, Goti E (unpubl.) MHC and mate choice in pheasants
19. Wittzell H, Bernot A, Auffray C, Zoorob R (1999) Concerted evolution of two Mhc class II B loci in pheasants and domestic chickens. Mol Biol Evol 16(4): 479-490.
20. Wittzell H, von Schantz T, Zoorob R, Auffray C (1994) Molecular characterization of three Mhc class II B haplotypes in the ring-necked pheasant. Immunogenetics 39(6): 395-403.
21. Hosomichi K, Shiina T, Suzuki S, Tanaka M, Shimizu S, Iwamoto S, Hara H, Yoshida Y, Kulski JK, Inoko H, Hanzawa K (2006) The major histocompatibility complex (Mhc) class IIB region has greater genomic structural flexibility and diversity in the quail than the chicken. BMC Genomics 7: 322.
22. Shimizu S, Shiina T, Hosomichi K, Takahashi S, Koyama T, Onodera T, Kulski JK, Inoko H (2004) MHC class IIB gene sequences and expression in quails (Coturnix japonica) selected for high and low antibody responses. Immunogenetics 56(4): 280-291.
23. Hosomichi K, Shiina T, Shimizu S, Inoko H, Hanzawa K (unpubl.) Characterization of Mhc class II B genes expressed in the chicken and quail immunological organs
24. Hosomichi K, Miller MM, Goto RM, Wang Y, Suzuki S, Kulski JK, Nishibori M, Inoko H, Hanzawa K, Shiina T (2008) Contribution of mutation, recombination, and gene conversion to chicken MHC-B haplotype diversity. J Immunol 181(5): 3393-3399.
25. Shiina T, Briles WE, Goto RM, Hosomichi K, Yanagiya K, Shimizu S, Inoko H, Miller MM (2007) Extended Gene Map Reveals Tripartite Motif, C-Type Lectin, and Ig Superfamily Type Genes within a Subregion of the Chicken MHC-B Affecting Infectious Disease. J Immunol 178(11): 7162-7172.
26. Burri R, Hirzel HN, Salamin N, Roulin A, Fumagalli L (2008) Evolutionary patterns of MHC class II B in owls and their implications for the understanding of avian MHC evolution. Mol Biol Evol 25(6): 1180-1191.
27. Nishibori M, Tsudzuki M, Yamamoto Y (unpubl.) Characterization of pseudogenes designated Y-Lb III in chicken MHC class II (B-L) genes
28. Soria LA, Iglesias GM, Jar AM, Miquel MC, Lopez OJ (unpubl.) Genetic polymorphism in the MHC B-L locus of Camperos, a mixed breed of broilers
