# Supplementary material for: MHC Class IIB Exon 2 Polymorphism in the Grey Partridge (Perdix perdix) Is Shaped by Selection, Recombination and Gene Conversion
Source: PLoS One. 2013 Jul 23;8(7):e69135. doi: 10.1371/journal.pone.0069135 (PMC3720538; doi:10.1371/journal.pone.0069135)
Supplement: File S3 — Posterior means of ω for each codon of MHCIIB exon 2 in the Grey partridge, calculated as the average of ω over the 11 site classes, weighted by the posterior probabilities under the random-sites model M8 (β and ω). The posterior probabilities were computed by the Bayes empirical Bayes procedure in the program CodeML implemented in the PAML3.14 package. (DOCX) [file pone.0069135.s003.docx]

**Supporting Information S3: Posterior means of ω for each codon of MHCIIB exon 2 in the Grey partridge, calculated as the average of ω over the 11 site classes, weighted by the posterior probabilities under the random-sites model M8 (β and ω).**

**Article title:** MHC class IIB exon 2 polymorphism in the Grey partridge (*Perdix perdix*) is shaped by selection, recombination and gene conversion

**Journal name:** PLOS ONE

**Authors:** Promerová M., Králová T., Bryjová A., Albrecht T. and Bryja J.

**Corresponding author:** Josef Bryja; e-mail: bryja@brno.cas.cz


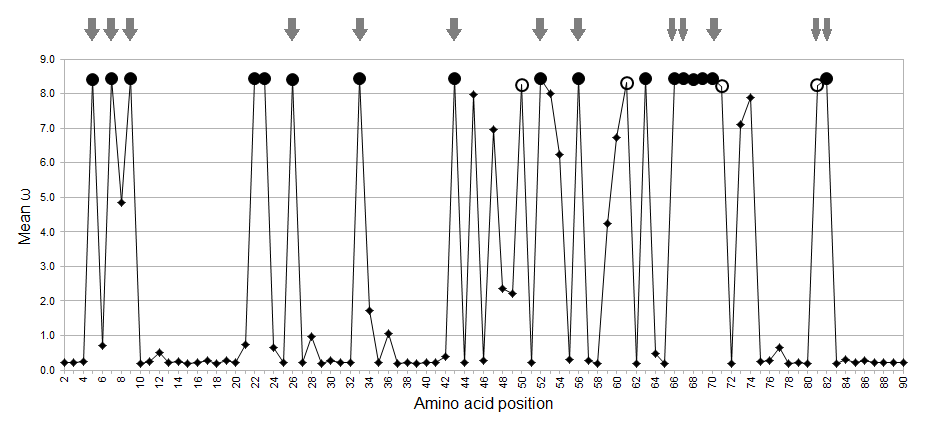


Posterior means of ω (dN/dS) for each codon of MHCIIB exon 2 in the Grey partridge, calculated as the average of ω over the 11 site classes, weighted by the posterior probabilities under the random-sites model M8 (β and ω). The posterior probabilities were computed by the Bayes empirical Bayes procedure in the program CodeML implemented in the PAML3.14 package. Full circles indicate sites under positive selection with probability >99%, empty circles mark sites positively selected with probability >95%. The arrows show those positively selected sites of the Grey partridge, which correspond to the PBR residues in humans (Brown et al. 1993).

**Reference**

Brown JH, Jardetzky TS, Gorga JC, Stern LJ, Urban RG, Strominger JL, Wiley DC (1993) Three-dimensional structure of the human class II histocompatibility antigen HLA-DR1. Nature 364: 33-39. doi: 10.1038/364033a0
